# Relationship between Stroke Volume and Pulse Pressure during Blood Volume Perturbation: A Mathematical Analysis

**DOI:** 10.1155/2014/459269

**Published:** 2014-05-20

**Authors:** Ramin Bighamian, Jin-Oh Hahn

**Affiliations:** Department of Mechanical Engineering, University of Maryland, 2181 Glenn L. Martin Hall, College Park, MD 20742, USA

## Abstract

Arterial pulse pressure has been widely used as surrogate of stroke volume, for example, in the guidance of fluid therapy. However, recent experimental investigations suggest that arterial pulse pressure is not linearly proportional to stroke volume. However, mechanisms underlying the relation between the two have not been clearly understood. The goal of this study was to elucidate how arterial pulse pressure and stroke volume respond to a perturbation in the left ventricular blood volume based on a systematic mathematical analysis. Both our mathematical analysis and experimental data showed that the relative change in arterial pulse pressure due to a left ventricular blood volume perturbation was consistently smaller than the corresponding relative change in stroke volume, due to the nonlinear left ventricular pressure-volume relation during diastole that reduces the sensitivity of arterial pulse pressure to perturbations in the left ventricular blood volume. Therefore, arterial pulse pressure must be used with care when used as surrogate of stroke volume in guiding fluid therapy.

## 1. Introduction

Stroke volume (SV) is the volume of blood pumped out by the heart to the arterial tree. It is known to be highly correlated with cardiac function in that it typically decreases in the presence of diseases such as cardiogenic shock [1], hemorrhage [2], sepsis [3], spinal cord injury [4], and hypothyroid [5]. It is also an important determinant of cardiac output, which is modulated by the demand for oxygen delivery to the tissues in the body [6] and the capacitance of the arteriovenous system [7]. Regarding its clinical applications, the interpretation of SV (or correspondingly cardiac output) can help caregivers to better understand the complex pathophysiological alterations in the critical illness, thereby helping to avoid deleterious effects of inotropic therapy [8], potentially harmful effects of vasopressor agents [9], and the detrimental edema in fluid administration [10].

Despite its clinical significance, SV has not been widely utilized for routine diagnostic and therapeutic purposes due to the difficulty in its measurement [11]. In fact, most state-of-the-art methods to directly measure SV (e.g., thermodilution technique and bioimpedance method) are invasive, expensive, and/or uncomfortable and necessitate trained experts for reliable measurement [12–15].

To exploit SV in clinical applications without encountering the problems listed above, there have been numerous efforts to indirectly estimate SV from minimally invasive or noninvasive arterial circulatory measurements, which are collectively called the pulse wave analysis (PWA) methods [16–19]. In a typical PWA method, arterial blood pressure (BP) and/or flow signals are analyzed via cardiovascular models [20–22], signal processing techniques [23, 24], feature extraction techniques [25], and so on.

In one of its simplest form, PWA is based on the assumption that SV is proportional to arterial pulse pressure (hereafter called pulse pressure (PP)) [16–19]. In fact, there are many existing evidences supporting this assumption [20, 21, 26]. Due to this reason, PP has been widely used as a convenient surrogate of SV during diagnostic and therapeutic procedures, such as fluid therapy [27], ventricular resynchronization therapy [28], and vasopressor/inotrope therapy [29].

Some recent experimental investigations suggest that although SV and PP are proportionally correlated during blood volume perturbation, the relationship may not be strictly linear, and PP may underestimate SV in response to blood volume changes [27, 30, 31]. It is possible that the underestimation of SV during fluid therapy may potentially require substantial correction for dosage regimen, since brute-force fluid administration based on linear SV-PP assumption is likely suboptimal. Indeed, the essential challenge in fluid therapy is to avoid the administration of too little or too much volume, since there is a relatively narrow range for safe fluid therapy and both overload and underhydration can adversely affect the patient outcome. In fact, it has been shown that patients receiving proper fluid therapy, compared with those receiving restricted fluid regimens due to underestimation of SV, have more than 50% fewer complications and shorter length of hospital stay [32]. In order for PP to be used as a reliable surrogate of SV during fluid therapy, the relationship between SV and PP in response to blood volume changes must be clearly understood. The goal of this study was to unveil the mechanisms underlying the relation between pulse pressure and stroke volume based on a systematic mathematical analysis in order to elucidate how pulse pressure and stroke volume respond to a perturbation in blood volume and validate our analysis with experimental data.

This paper is organized as follows. In Section 2, the left-ventricular pressure-volume loop is introduced as a framework for our analysis. In Section 3, the responses of SV and PP to blood volume perturbation are analyzed, based on which the relationship between SV and PP during blood volume change is elucidated. The mathematical analysis is compared with experimental data in Section 4.

## 2. Left-Ventricular Pressure-Volume Framework

**Figure 1:**
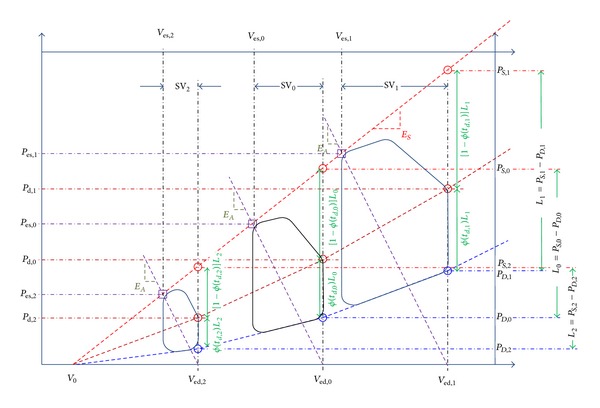
Left ventricular pressure-volume loop for different end-diastolic volumes.

We use the left ventricular (LV) pressure-volume loop (*P*-*V* loop) framework [33] to mathematically analyze how changes in SV and PP are related during volume perturbation. In the context of LV *P*-*V* loop, the so-called “maximum” LV pressure [33–35] is given by the weighted average of end-systolic and end-diastolic pressures:

(1) $$\begin{array}{c} P_{\text{LV}}^{\max}=\phi \left(t\right)P_{S}\left(V\left(t\right)\right)+\left(1-\phi \left(t\right)\right)P_{D}\left(V\left(t\right)\right), \end{array}$$

where *ϕ*(*t*) is the activation function [33, 35, 36] and *P*_*S*_ and *P*_*D*_ are the pressures corresponding to end-systolic and end-diastolic *P*-*V* relationships at a LV volume *V*(*t*) [33, 35]. *P*_*S*_ and *P*_*D*_ are given by (see red and blue dashed lines in Figure 1)

(2) PS(V(t))=ES(V(t)−V0),PD(V(t))=B[eA(V(t)−V0)−1],

where *E*_*S*_ is the end-systolic LV elastance, *A* and *B* are constants specifying the end-diastolic *P*-*V* relationship, and *V*_0_ is the LV volume corresponding to zero LV pressure [33, 35, 36].

In Section 3, we exploit the above well-established mathematical model to elucidate the relationship between the changes in SV and PP during volume perturbation.

## 3. Relationship between SV and PP during Volume Perturbation

In this study, the mechanisms underlying the relation between SV and PP during volume perturbation are elucidated as follows. First, we show how SV changes in response to changes in end-diastolic volume (due to volume perturbation). Second, we show how PP changes in response to changes in end-diastolic volume. Using these two results, we finally explain how PP changes relative to SV in response to changes in end-diastolic volume.

### 3.1. SV Response to Volume Perturbation

In the context of *P*-*V* loop, SV can be computed from end-diastolic volume as follows. By definition, SV is given by the difference between end-diastolic and end-systolic volumes:

(3) $$\begin{array}{c} \delta V=V_{\text{ed}}-V_{\text{es}}=V\left(t_{\text{ed}}\right)-V\left(t_{\text{es}}\right), \end{array}$$

where *V*_ed_ = *V*(*t*_ed_) and *V*_es_ = *V*(*t*_es_) are end-diastolic and end-systolic volumes and *t*_ed_ and *t*_es_ are the time instants corresponding to end-diastole and end-systole, respectively. Alternatively, SV is given from mean arterial pressure (MAP) as follows:

(4) $$\begin{array}{c} \delta V=\frac{P_{m}}{R}T, \end{array}$$

where *P*_*m*_ is MAP, *R* is total peripheral resistance (TPR), and *T* is heart period. At end-systole (*t* = *t*_es_), the *P*-*V* loop intersects with the systolic *P*-*V* relationship *P*_*S*_ = *E*_*S*_(*V*(*t*) − *V*_0_) [33, 35], where *P*_*S*_ = *P*_es_ and *V*(*t*) = *V*(*t*_es_) = *V*_es_. Therefore, we have

(5) $$\begin{array}{c} P_{\text{es}}=E_{S}\left(V_{\text{es}}-V_{0}\right). \end{array}$$

On the other hand, since end-systolic pressure is typically very close in value to MAP [37, 38], we have, from (4),

(6) $$\begin{array}{c} \delta V=V_{\text{ed}}-V_{\text{es}}≅\frac{P_{\text{es}}}{R}T. \end{array}$$

Combining (5) and (6) yields the following expression for *V*_es_:

(7) $$\begin{array}{c} V_{\text{es}}=\frac{E_{A}}{E_{S}+E_{A}}V_{\text{ed}}+\frac{E_{S}}{E_{S}+E_{A}}V_{0}, \end{array}$$

where *E*_*A*_ = *R*/*T* is called the arterial elastance [33, 35, 38]. Therefore, SV can be computed from end-diastolic volume as

(8) $$\begin{array}{c} \delta V=V_{\text{ed}}-V_{\text{es}}=\frac{E_{S}}{E_{S}+E_{A}}\left(V_{\text{ed}}-V_{0}\right). \end{array}$$

Thus, SV is related to end-diastolic volume by the proportionality constant *E*_*S*_/(*E*_*S*_ + *E*_*A*_), which depends on LV and arterial elastances. Therefore, it can be concluded that a change in end-diastolic volume caused by volume perturbation results in a change in SV whose magnitude is linearly proportional to that of end-diastolic volume,* if LV and arterial elastances remain constant* during volume perturbation. In Figure 1, this can be illustrated as the linear proportionality between the triangles defined by (*V*_ed,*j*_, 0), (*V*_0_, 0), and (*V*_es,*j*_, *P*_es,*j*_), *j* = 0,1, 2: as long as *E*_*S*_ and *E*_*A*_ remain constant, SV ( = *V*_ed,*j*_ − *V*_es,*j*_ = *P*_es,*j*_cot^−1^⁡*E*_*A*_ = (*E*_*S*_ *E*_*A*_/(*E*_*S*_ + *E*_*A*_))(*V*_ed,*j*_ − *V*_0_)cot^−1^⁡*E*_*A*_) is proportional to the end-diastolic volume ( = *V*_ed,*j*_ − *V*_0_).

### 3.2. PP Response to Volume Perturbation

To understand the PP response to volume perturbation, we first analyze the responses of end-systolic and diastolic (DP) pressures to changes in end-diastolic volume and then show the response of PP by formulating it to the difference between end-systolic pressure response and DP response. The rationale for using end-systolic pressure and DP rather than systolic pressure (SP) and DP is because, in contrast to end-systolic pressure and DP which always occur at end-systolic and end-diastolic volumes (see Figure 1), the value of volume on the *P*-*V* loop where SP occurs is not straightforward to specify. It will be demonstrated that PP can be, at least approximately, obtained from end-systolic pressure and DP by assuming that end-systolic pressure is typically very close in value to MAP.

At diastole (*t* = *t*_*d*_ where *t*_*d*_ is the time instant corresponding to DP), the maximum LV pressure is equal to DP, and LV volume is equal to end-diastolic volume (*V*_ed_). Therefore, (1) reduces to

(9) Pd(Ved)ϕ(td)PS(Ved)+(1−ϕ(td))PD(Ved)=ϕ(td)ES(Ved−V0) +(1−ϕ(td))B[eA(Ved−V0)−1].

For simplicity of analysis, assume that *t*_*d*_ relative to *T* remains constant during volume perturbation (see Section 3.4 for what happens if this assumption is relaxed). Then, it is obvious from (9) that, for a given value of end-diastolic volume, DP is determined as the weighted average of end-systolic and end-diastolic pressures corresponding to that end-diastolic volume:

(10) Pd(Ved)σPS(Ved)+(1−σ)PD(Ved)=σES(Ved−V0)+(1−σ)B[eA(Ved−V0)−1],

where *σ* = *ϕ*(*t*_*d*_) is constant* if t*_*d*_ *relative to T remains constant*. Now, if we note that the end-systolic *P*-*V* relationship, *E*_*S*_(*V*_ed_ − *V*_0_), is linear in *V*_ed_, whereas the end-diastolic *P*-*V* relationship, *B*[*e* ^*A*(*V*_ed_−*V*_0_)^ − 1], is exponential in *V*_ed_, and also that *P*_*d*_(*V*_ed_) is simply the weighted average between the two, it can be concluded that the rate of change in DP increases as end-diastolic volume increases (see Figure 1). This is illustrated in Figure 1 by the brown dashed line connecting *P*_*d*,*j*_ = *P*_*d*_(*V*_ed,*j*_), *j* = 0,1, 2, whose slope becomes steeper as end-diastolic volume increases.

The response of end-systolic pressure to changes in end-diastolic volume can be obtained by combining (5) and (7), which yields

(11) $$\begin{array}{c} P_{\text{es}}\left(V_{\text{ed}}\right)=E_{S}\left(V_{\text{es}}-V_{0}\right)=\frac{E_{S}E_{A}}{E_{S}+E_{A}}\left(V_{\text{ed}}-V_{0}\right). \end{array}$$

Thus, end-systolic pressure is related to end-diastolic volume by the proportionality constant *E*_*S*_ *E*_*A*_/(*E*_*S*_ + *E*_*A*_), which depends on LV and arterial elastances. Therefore, it can be concluded that end-systolic pressure is linearly proportional to end-diastolic volume* if LV and arterial elastances remain constant* during volume perturbation.

To relate end-systolic pressure and DP to PP, we use a widely accepted relationship between SP, MAP, and DP: *P*_*m*_≅*P*_*d*_ + (1/3)(*P*_*s*_ − *P*_*d*_). As for Section 3.1, if we assume that end-systolic pressure is very close to MAP (*P*_es_ ≈ *P*_*m*_), we get the following relationship between PP, end-systolic pressure, and DP:

(12) Pes≅Pd+13(Ps−Pd)⟶Pp=Ps−Pd≅3(Pes−Pd)

which indicates that PP is linearly proportional to the difference between end-systolic pressure and DP.

Finally, combining the conclusions drawn from (10)–(12), we can conclude that the rate of change in PP decreases as end-diastolic volume increases, because the rate of change in DP becomes steeper than that in end-systolic pressure as end-diastolic volume increases (see Figure 1). This can be illustrated in Figure 1 as follows: as long as LV and arterial elastances as well as *ϕ*(*t*_*d*_) remain constant, the rate of change in *P*_es,*j*_ − *P*_*d*,*j*_ decreases with an increase in end-diastolic volume (see the left vertical axis), since the difference between the slopes of red (*P*_es_) and brown (*P*_*D*_) lines decreases as end-diastolic volume increases.

### 3.3. Relationship between SV and PP

**Figure 2:**
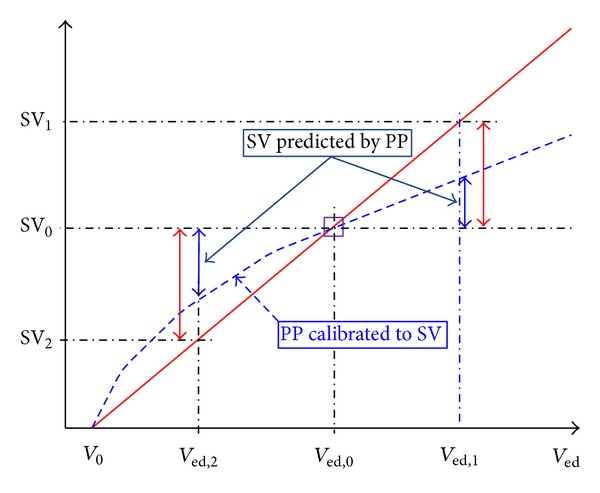
Relationship between SV and PP.

The analyses performed in Sections 3.1 and 3.2 indicate that, under the assumption that (1) both LV and arterial elastances as well as *t*_*d*_ relative to *T* remain constant during volume perturbation, and (2) end-systolic pressure is close in value to MAP, SV shows constant proportionality to end-diastolic volume as indicated in (8) (i.e., it is a linear function of end-diastolic volume). In contrast, PP exhibits decreasing proportionality to end-diastolic volume with an increase in end-diastolic volume, thereby decreasing the rate of change in PP response to end-diastolic volume as it increases (in other words, PP shows a gradually decreasing slope when it is plotted against end-diastolic volume). Since SV and PP exhibit constant versus decreasing slopes against end-diastolic volume, respectively, the relationship between SV and PP is concave towards SV. Therefore, SV and PP are not linearly proportional to each other, and the rate of change in PP is not a good quantitative indicator of the rate of change in SV. In fact, our analyses suggest that the rate of change in PP underestimates the rate of change in SV in the neighborhood of a given operating end-diastolic volume (see Figure 2). Indeed, Figure 2 illustrates that the slope of SV with respect to end-diastolic volume is steeper than that of PP around the vicinity of an operating end-diastolic volume.

### 3.4. Relaxation of Assumptions

In our analysis, we made the following assumptions: during changes in end-diastolic volume due to volume perturbation, (i) the time instant corresponding to DP relative to the heart period (*t*_*d*_/*T*) is constant (A1); (ii) end-systolic pressure is close in value to MAP (A2); and (iii) LV and arterial elastances remain constant (A3). In this section, these assumptions are relaxed and their effects are incorporated to the conclusion drawn in Section 3.3.

#### 3.4.1. Relaxation of Assumption (A1)

It has been suggested that the shape of the activation function *ϕ*(*t*) is highly consistent among different individuals that is, its inter-individual variability is small [39, 40]. However, the timing values associated with cardiac events, for example, diastole (*t* = *t*_*d*_) may be subject to change due to mechanisms such as baroreflex. This may invalidate the assumption (A1) above. Thus, it is worthwhile to examine how the timing-related variability in *ϕ*(*t*) alters the relationship between SV and PP.

It is obvious from (8) that SV is not influenced by *ϕ*(*t*). In addition, (10)–(12) indicate that PP is related to *ϕ*(*t*) only via DP but not via end-systolic pressure. So, uncertainty in *ϕ*(*t*) affects the relationship between SV and PP by altering DP (which occurs at *t* = *t*_*d*_). Consequently, variability in the time instant corresponding to diastole (*t* = *t*_*d*_) turns out to be the main parameter to be analyzed. In this study, we perform sensitivity analysis to quantitatively assess how significantly the relationship between SV and PP is altered by the variability in *t*_*d*_. Using (10)–(12), PP can be rewritten as follows:

(13) Pp=Ps−Pd≅3(Pes−Pd)=3{ESEAES+EA(Ved−V0)  −[σPS(Ved)+(1−σ)PD(Ved)]}.

Then, the sensitivity of PP with respect to *t*_*d*_/*T* is given by

(14) ∂Pp∂(td/T)≅−3∂Pd∂(td/T)=−3∂σ∂(td/T)[PS(Ved)−PD(Ved)].

In (14), [*P*_*S*_(*V*_ed_) − *P*_*D*_(*V*_ed_)] does not depend on *t*_*d*_; it is a function of *V*_ed_ only. Since the term ∂*σ*/∂(*t*_*d*_/*T*) (i.e., the sensitivity of the activation function with respect to *t*_*d*_/*T*) is always positive [34], it can be concluded that PP decreases as *t*_*d*_/*T* increases.

#### 3.4.2. Relaxation of Assumption (A2)

The effect of discrepancy between MAP and end-systolic pressure on the relationship between SV and PP can be examined as follows. It is clear from (8) and (12) that only PP but not SV is affected. The error in PP (${\tilde{P}}_{p}$) due to the difference between MAP and end-systolic pressure is given by

(15) P~p=3(Pes−Pd)−3(Pm−Pd)=3P~m,

where ${\tilde{P}}_{m}=P_{\text{es}}-P_{m}$. Thus, an error in MAP (caused by approximating it to end-systolic pressure) is propagated to the PP error with an amplification factor of 3 (e.g., 1% error in MAP results in 3% error in PP), which can be deleterious if the MAP error is large. However, the absolute magnitude of alteration in PP due to the discrepancy between MAP and end-systolic pressure is not expected to be significant, since MAP is indeed close in value to end-systolic pressure over a wide range of physiologic conditions [37, 38].

#### 3.4.3. Relaxation of Assumption (A3)

First, the effect of arterial elastance on the responses of end-systolic pressure, PP, and SV anticipated due to the changes in end-diastolic volume is summarized in Table 1. In theory, TPR and the heart rate (the inverse of heart period) are altered by the autonomic baroreflex in response to alterations in *V*_ed_ [41, 42]. Specifically, an increase in end-diastolic volume results in a decrease in TPR and heart rate, whereas they increase to a decrease in end-diastolic volume [41, 42]. Therefore, the arterial elastance decreases during an increase in end-diastolic volume, which then yields a decrease in end-systolic pressure (with respect to its value predicted under constant arterial elastance) via a decrease in *E*_*S*_ *E*_*A*_/(*E*_*S*_ + *E*_*A*_). This then results in a decrease in PP, since DP is not affected by the arterial elastance. On the other hand, a decrease in arterial elastance yields an increase in SV (again, with respect to its value predicted under constant arterial elastance) via an increase in *E*_*S*_/(*E*_*S*_ + *E*_*A*_). Therefore, should there be any notable impact of end-diastolic volume on arterial elastance, the underestimation of SV based on PP will be exacerbated during an increase in end-diastolic volume, for example, during fluid therapy. In contrast, it can be deduced, based on the reasoning consistent with the above, that PP and SV will, respectively, increase and decrease from their values predicted under constant arterial elastance if end-diastolic volume decreases. Thus, the underestimation of SV based on PP will be alleviated during a decrease in end-diastolic volume, for example, hemorrhage.

Second, the effect of LV elastance on the responses of end-systolic pressure, PP, and SV anticipated due to the changes in end-diastolic volume is summarized in Table 2. Similarly to TPR and heart rate, LV elastance is altered by the autonomic baroreflex in response to alterations in *V*_ed_ [43]. In particular, LV elastance typically decreases if end-diastolic volume increases, and it increases if end-diastolic volume decreases [43]. It can then be shown that both *E*_*S*_ *E*_*A*_/(*E*_*S*_ + *E*_*A*_) and *E*_*S*_/(*E*_*S*_ + *E*_*A*_) decrease in response to an increase in end-diastolic volume. Consequently, an increase in end-diastolic volume will result in a decrease in end-systolic pressure and SV (with respect to their values predicted under constant LV elastance), whereas a decrease in end-diastolic volume will result in an increase in end-diastolic pressure and SV (again, with respect to their values predicted under constant LV elastance). In addition, DP is also affected by the LV elastance, because a change in LV elastance alters the value of *P*_*S*_(*V*_ed_) (see Figure 1). Therefore, the effect of LV elastance on PP can be elucidated by combining its impacts on end-systolic pressure and DP. To quantify the effect of LV elastance on PP, consider the following equations for end-systolic pressure and DP in response to a perturbation on LV elastance:

(16) (Pes+ΔPes)=(ES+ΔES)EA(ES+ΔES)+EA(Ved−V0),(Pd+ΔPd)=σ(ES+ΔES)(Ved−V0) +(1−σ)B[eA(Ved−V0)−1].

Thus, alterations in end-systolic pressure and DP can be written as follows:

(17) ΔPes=[(ES+ΔES)EA(ES+ΔES)+EA−(ES)EA(ES)+EA](Ved−V0)≈(EAES+EA)2ΔES(Ved−V0),ΔPd=σΔES(Ved−V0),

where the expression for Δ*P*_es_ was simplified using the Taylor series expansion. Consequently, the alteration of PP due to a perturbation in LV elastance can be quantified as follows:

(18) ∂Pp∂ES=3[(EAES+EA)2−σ](Ved−V0).

So, whether PP increases or decreases depends on the sign of [(*E*_*A*_/(*E*_*S*_+*E*_*A*_))^2^ − *σ*]. Though not definitive, it can be shown numerically that [(*E*_*A*_/(*E*_*S*_+*E*_*A*_))^2^ − *σ*] takes negative values over the space of physiologically nominal parameter values. Therefore, should there be any notable impact of end-diastolic volume on LV elastance, the underestimation of SV based on PP will be alleviated during an increase in end-diastolic volume, for example, during fluid therapy. In contrast, the underestimation of SV based on PP will be exacerbated during a decrease in end-diastolic volume, for example, hemorrhage.

### 3.5. Simulation Study

**Figure 3:**
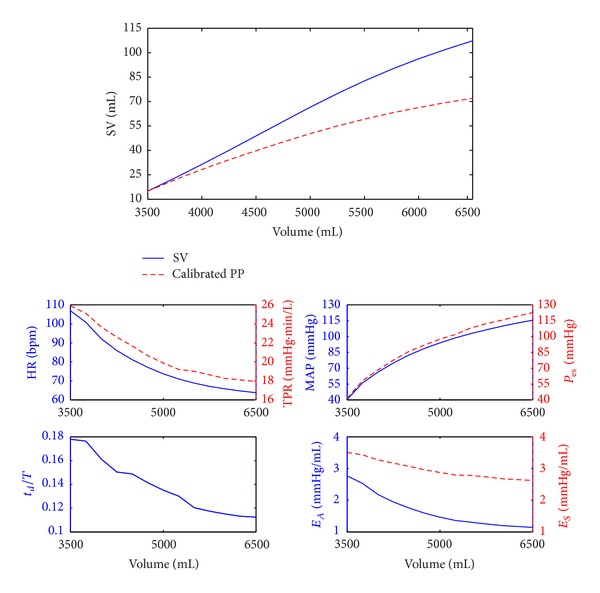
A representative result of SV, BP, and baroreflex responses to a wide range of perturbation in blood volume (3.5 L–6.5 L).

To numerically examine the results of the analysis in this section, a simulation model developed by Ursino [44] and Ursino and Magosso [45, 46] was used to create SV and PP responses to a wide range of hypothetical volume perturbations. The model includes a time-varying elastance model of the heart, arterial and venous vessels lumped into 12 compartments, and a nonlinear baroreflex feedback model. In the simulation model, blood volume was varied from 3.5 L to 6.5 L (with nominal volume of 5.0 L), and the corresponding BP and SV responses in the steady state were obtained. A representative result is shown in Figure 3, where PP has been scaled to SV so that their values at 3.5 L match.

First of all, the simulation result shown in Figure 3 makes sure that the change in PP underestimates that in SV. For example, the change in SV as predicted by the change in PP in response to the added blood volume of 3.0 L (from 3.5 L to 6.5 L) was only ~60% of the actual change in SV. Therefore, PP must not be used as a linear predictor of SV.

It is noted that the result shown in Figure 3 was obtained in the presence of realistic variability in *t*_*d*_/*T*, *E*_*A*_, and *E*_*S*_. Indeed, the baroreflex feedback responses in Figure 3 indicate that these parameters were subject to nonnegligible variability during blood volume perturbation. In particular, *t*_*d*_/*T* decreased by large amount in response to an increase in blood volume, which was attributed to a large decrease in HR (thus a large increase in *T*). Also, TPR as well as arterial and LV elastances decreased as blood volume increased, which was anticipated. Compared with LV elastance, however, the variability in arterial elastance was significantly larger due to large changes in HR and TPR.

**Figure 4:**
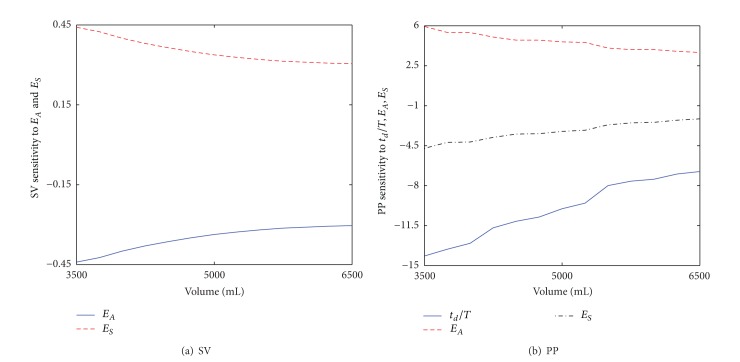
Sensitivity of SV and PP to *t*_*d*_/*T*, *E*_*A*_, and *E*_*S*_.

To quantitatively examine the effect of variability in *t*_*d*_/*T*, *E*_*A*_, and *E*_*S*_ on our analysis, the sensitivity of SV and PP to these parameters was computed and scrutinized (see Figure 4). Overall, the sensitivity of SV on *E*_*A*_ and *E*_*S*_ was very small (see Figure 4(a)). Also, it does not explicitly depend on *t*_*d*_/*T* as indicated by (8). Thus, we predicted that the assumptions (A1)–(A3) made in Section 3 would not affect SV. Indeed, simulated SV as shown in Figure 3 was very close in value to SV predicted from (8) under constants *E*_*A*_ and *E*_*S*_ (not shown). On the other hand, PP turned out to be largely affected by these parameters (see Figure 4(b)). Considering that the absolute amount of change in *E*_*A*_ was much larger than that in *E*_*S*_ (see Figure 3), it turned out that the effect of changes in *t*_*d*_/*T* and *E*_*A*_ on PP was dominant in comparison with the effect of change in *E*_*S*_. Now that the direction of changes in *t*_*d*_/*T* and *E*_*A*_ is the same (i.e., both decrease for positive volume perturbation but increase for negative volume perturbation) but their impact on PP is opposite (as indicated by opposite signs in sensitivity, see Figure 4(b)), it was observed that their effects were approximately canceled by each other. So, together with the observation that end-systolic pressure was consistently higher than MAP (see Figure 3), PP was overestimated based on (15). Summarizing all these observations, relaxation of the assumptions (A1)–(A3) made in Section 3 appears to further pronounce PP's underestimation of SV.

## 4. Experimental Data Analysis

To experimentally examine the validity of mathematical analysis conducted in this study, we analyzed a subset of *P*-*V* loop data collected in a previous study [47]. Data pertaining to 5 human subjects were analyzed, each of which had LV *P*-*V* loops associated with multiple LV volumes, electrocardiogram (ECG), and central aortic BP waveform. In each *P*-*V* loop, ECG was used to identify the beginning of diastole. The time instant at which LV pressure attains its maximum was regarded as the systolic peak (LV pressure = SP), from which the time instants corresponding to DP and end-systolic pressure were determined based on the time rate of change of LV pressure. Then, end-diastolic and end-systolic LV volumes were derived as average LV volume values during isovolumetric contraction and relaxation phases, respectively. SV was then determined by subtracting end-systolic volume from end-diastolic volume. PP was derived directly from the central aortic BP waveform.

**Figure 5:**
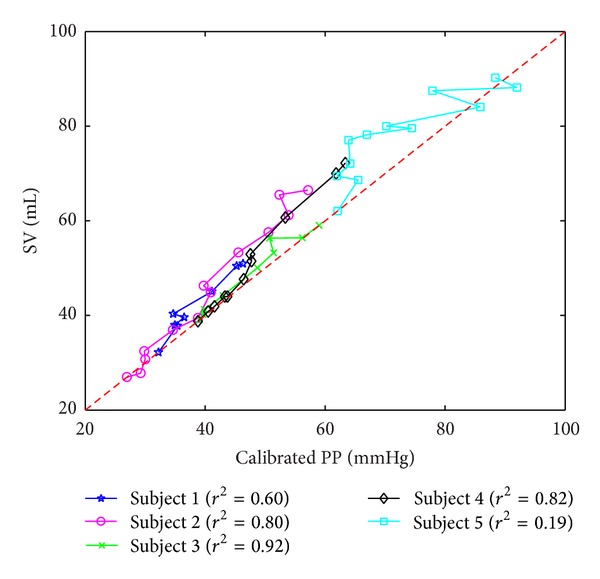
Experimental relation between SV and PP in humans.

Figure 5 shows the relation between SV and calibrated (at the smallest SV) PP obtained from the data. The pairs of SV and PP mostly lie above the red dashed line corresponding to *δV* = *P*_*p*_, meaning that PP indeed underestimates SV. On the average, the *r* ^2^ value between SV and calibrated PP was only 0.67, and the amount of change in SV was about 1.36 times larger than the amount of change in PP for a given perturbation in LV end-diastolic volume. It is also obvious in Figure 1 that the trend of underestimation was more significant as LV volume increased (especially in subjects 1, 2, 4, and 5, although in subject 5 outliers were observed due to noisy LV *P*-*V* loop measurement). All in all, observations from Figure 5 are highly consistent with the mathematical analysis conducted earlier in this study (Section 3).

**Figure 6:**
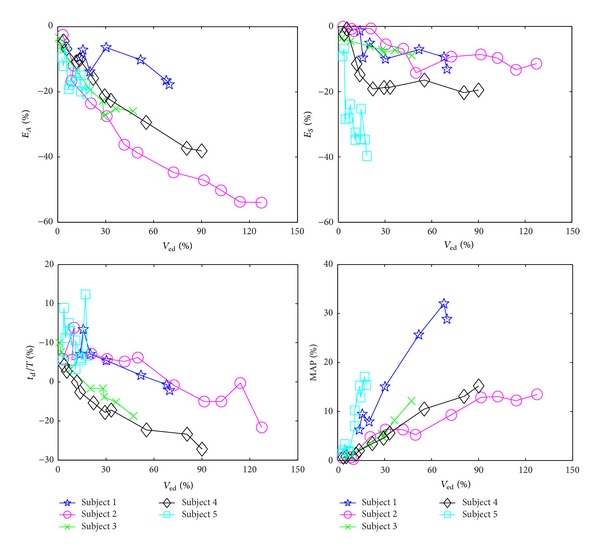
Behaviors of arterial (*E*_*A*_) and LV (*E*_*S*_) elastances, normalized DP time instant (*t*_*d*_/*T*), and MAP in response to perturbations in end-diastolic volume.

It is also worth mentioning that the experimental data indicated that (i) MAP and end-systolic pressure were very close to each other, and that (ii) the experimentally observed behaviors of arterial (*E*_*A*_) and LV (*E*_*S*_) elastances, normalized DP time instant (*t*_*d*_/*T*), and MAP in response to perturbations in end-diastolic volume were also consistent with the mathematical analysis conducted in Sections 3.4 and 3.5 (Figure 6). First, the difference between MAP and end-systolic pressure observed in the data was only 9 ± 4% of the end-systolic pressure. Second, the trends of *E*_*A*_, *E*_*S*_ and *t*_*d*_/*T* were all inversely proportional to end-diastolic volume, while MAP was proportional to end-diastolic volume. Third, comparing the amount of changes in *E*_*A*_, *E*_*S*_ and *t*_*d*_/*T*, the change in *E*_*A*_ dominated those in *E*_*S*_ and *t*_*d*_/*T* (see Figures 3 and 6), which, as discussed in Section 3.5, is the basis to justify that the assumptions (A1) and (A3) will not significantly affect the relation between SV and PP. Therefore, together with Figure 5, Figure 6 supports the validity of our mathematical analysis (see Section 3.3) to a large extent: (i) the underestimation of SV by PP is mainly due to the nonlinear LV *P*-*V* relation during diastole that ultimately reduces the sensitivity of PP to LV volume, and (ii) the assumptions made in Section 3.4 will not affect our analysis significantly.

## 5. Conclusion and Future Work

Pulse pressure has been observed to underestimate stroke volume in recent experimental studies, but the mechanisms underlying the relation between the two have not been clearly understood. In this study, we elucidated the mechanisms underlying the nonlinear dependence between SV and PP. In sum, the rate of change in PP decreases with end-diastolic volume, while SV depends linearly on end-diastolic volume. Therefore, PP underestimates SV. Considering that PP is frequently used as a direct surrogate of SV, this entails an important clinical implication: nonoptimal fluid therapy may result if there is no correction to PP to compensate for its nonlinear dependence on SV. In our opinion, the analysis conducted in this study may be useful for developing methods to enable such compensation in the follow-up studies.

## Figures and Tables

**Table 1:** Effect of arterial elastance on the responses of end-systolic pressure, PP, and SV.

	*R*	*T*	*E*_*A*_	*E*_*S*_ *E*_*A*_/(*E*_*S*_ + *E*_*A*_)	*E*_*S*_/(*E*_*S*_ + *E*_*A*_)	*P*_es_	*P*_*p*_	*δV*
*V*_ed_↑	↓	↑	↓	↓	↑	↓	↓	↑
*V*_ed_↓	↑	↓	↑	↑	↓	↑	↑	↓

**Table 2:** Effect of LV elastance on the responses of end-systolic pressure, PP, and SV.

	*E*_*S*_	*E*_*S*_ *E*_*A*_/(*E*_*S*_ + *E*_*A*_)	*E*_*S*_/(*E*_*S*_ + *E*_*A*_)	*P*_es_	*P*_*d*_	*P*_*p*_	*δV*
*V*_ed_↑	↓	↓	↓	↓	↓	↑	↓
*V*_ed_↓	↑	↑	↑	↑	↑	↓	↑
